# Prevalence of Cutaneous Leishmaniasis in Western Highlands in Yemen

**DOI:** 10.1155/2019/8248916

**Published:** 2019-02-28

**Authors:** Mohammed Musid Alkulaibi, Ahmed Mohamed Suleiman, Eltahir Awad Gasim Khalil, Maged Ahmed Al-Garadi

**Affiliations:** ^1^Department of Oral and Maxillofacial Surgery, Faculty of Dentistry, University of Khartoum, Khartoum, Sudan; ^2^Department of Oral and Maxillofacial Surgery, Faculty of Dentistry, Sana'a University, Sana'a, Yemen; ^3^Department of Clinical Pathology & Immunology Institute of Endemic Diseases, University of Khartoum, Khartoum, Sudan; ^4^Department of Veterinary Medicine, Faculty of Agriculture and Veterinary Medicine, Thamar University, Dhamar, Yemen

## Abstract

Leishmaniasis in Yemen is still not fully investigated nor well studied. Recently, outbreaks of cutaneous leishmaniasis (CL) in western highland were declared. However, there are no reports concerning the disease and the circulating species in the region. The aim of this study was to determine the prevalence of cutaneous leishmaniasis in Utmah district located in Western Highlands in Yemen. A cross-sectional survey was carried out at those highlands. For the survey, 1165 participants were subjected to Leishmanin Skin Test (LST) accompanied with direct interviews and physical examination. The overall prevalence of cutaneous leishmaniasis in the district was 18.5% and the cutaneous leishmaniasis (CL) was more frequent in the escarpments with a prevalence of 37%, including 5.5% for active lesion and 31.5% for scar of healed lesions. Children under the age of 16 years old comprised most of the CL cases (76.3%). The escarpments of western highlands in Yemen were hyperendemic areas for CL and the infection was more prevalent in children.

## 1. Introduction

Cutaneous leishmaniasis (CL) is an endemic disease in the Middle East and North Africa including Yemen. Sudan, Egypt, Libya, Jordan, Tunisia, and Palestine are endemic mainly for the zoonotic cutaneous leishmaniasis (ZCL) while Syria, Saudi Arabia, Iraq, and Iran are endemic for both ZCL and anthroponotic cutaneous leishmaniasis (ACL) [1]. In the neighboring countries of Yemen, starting from Saudi Arabia, the recent documented studies reveal that the ACL is the prevalent form of CL in western highland, the same highland that extended through Yemen in the western region, the reported incidence ranging from 0.1% to 0.4%, and the infection is prevalent at escarpment land while the ZCL is prevalent in central and eastern provinces [2–6]. In Oman, reports showed that the prevalent form of CL is the zoonotic form which is mainly caused by L. major. In Ethiopia, all forms of CL including diffused cutaneous leishmaniasis (DCL) are existed and the overall prevalence is ranging from 4% to 44% [7–10]. In Sudan where VL is hyperepidemic, CL is widely distributed in Kordufan, Darfur, and north of Khartoum [5, 11, 12]. Along the River Nile, north of Khartoum, LST survey revealed high prevalence of leishmaniasis accounting 94% of population [13]. In Jordan, CL have been reported in Aqaba, north Agwar, and south Shuneh during a period from 2004 to 2008. An average incidence rate of CL was estimated in Jordan Valley revealing 73/100,000 person-year [14, 15]. In Syria, CL is documented in Aleppo province since a long period of time and known as Aleppo boil [16]. WHO recognized this region as one of the highest endemic regions in world revealing incidence cases of 73.8/100,000 person-years [14, 15]. Recently, it was reported that the incidence raised up to 100,000 cases per year and political war in the country since 2011 was reported to be the attributed cause [17]. In Iraq, the incidence rates of CL is 5/100,000 person-years [18]; in 1992, the number of CL peaked at 45.5 cases per 100,000 and, in 2008, an outbreak of CL was reported in Diwania and Rahmania province where 300 cases and 400 cases of CL were registered, respectively [18]. In Iran, the reported prevalence ranged from 0.7 to 4.7% [19, 20].

In Yemen, CL has been reported at northwestern, southwestern, and central highlands [21–23]. Although the first report about CL in Yemen has been documented in 1933, little about leishmaniasis epidemiology is known. In the northwestern highlands at Hajjah governorate, Khatri et al. [24] and Mogalli et al. [25] reported that the CL appeared to be endemic and outbreak was going on the region. In their recent study, Khatri et al. [24] reported that L.* tropica* was the dominant causative species accounting for 95% of cases while L.* donovani* and L.* infantum* represented a low percentage (4.1%) and 1.9% was for atypical molecular patterns. In the southwestern highlands at Lahj and Taiz governorates, Mugbil et al. [26] and Alharazi et al. [27] reported that CL was endemic disease mainly in the rural regions [26, 27]. In the central highlands at Al-Bayda province, Al-Kamel reported that the CL represented 4.11% of skin diseases [28, 29]. However, in 1989, Rioux et al. [30] reported L.* donovani* and L.* infantum* being the causative species for visceral leishmaniasis (VL) and L.* tropica* being the causative agent for CL in Tehama city in Yemen. Alvar et al. [31] in their worldwide and global estimates of Leishmaniasis presented a total of 603 reported CL cases/year from 2005 to 2009 with estimated annual CL incidences of 3000-6000 cases. All the above mentioned studies in Yemen were based on data of patients attending clinics and no survey was done to estimate the actual prevalence [32]. Nowadays, new foci of CL emerged in Utmah distract, western highlands in Yemen, as declared by Ministry of Public health and Population (MPHP). Accordingly, the present study aimed to evaluate the epidemiologic profile of CL in these highlands.

## 2. Materials and Methods

### 2.1. Study Population

**Figure 1:**
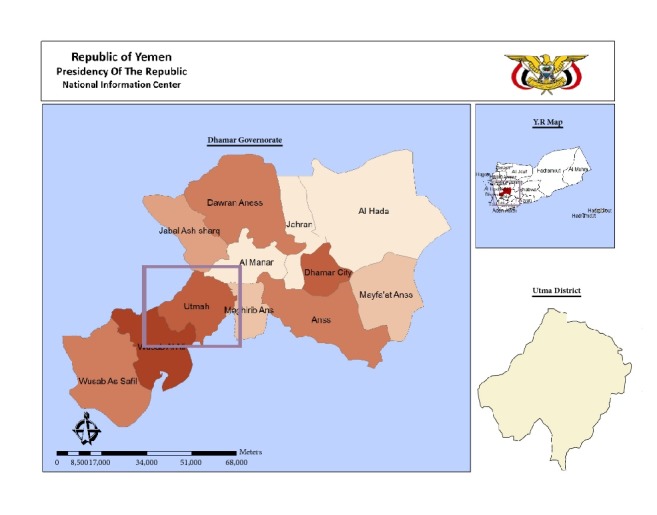
Yemen map showing Dhamar governorate and Utmah district.

Utmah district is part of the western highland that extends from the north, adjacent Saudi Arabia to the south near the costal line of the Arab Sea. Precisely, the district is located in the middle portion of the highland, 60 Km to the west of Dhamar city (Figure 1). The study population was around 145284 subjects. Of them, 68082 were males and 77202 were females as reported by Yemen National Census of 2004. The people are living in 57 localities (villages) and depend on agriculture and grazing of domesticate animals for their life. Housewives, children, and elderly individuals are staying in the district looking after farms and animals while most of adult males are outside the district as soldiers or careers in the surrounding cities. People are more concentrated in the plateau lands representing 60% of the study population while the rest of the population in the mountainous lands.

Ethical Committee at Disease Control and Surveillance, Ministry of Health, Yemen approved the study (Ref: 10/462). Participants were informed about the objectives, methodology, possible risks, and benefits of the study and they were asked to sign a written informed consent [33] or to give a verbal consent. Verbal agreement to participate was obtained only from subjects who were illiterate and could not read and sign the written consent. Detailed explanation about the study was provided orally and subjects were allowed to ask any questions and the related answers were given. At the end, the same statement of the written consent was read orally before subjects and they gave their consent to participate. For children who were involved in this study, parents' consent was obtained accordingly. The survey started at the beginning of March 2015 until the end of July 2015.

### 2.2. Sample Size

Based on clustering sample technique 1160 participants of the study population were recruited and divided into 58 clusters; each cluster contains 20 subjects. The clusters were distributed over the localities of Utmah district. Participants who had mental or medical problem(s) or infants and participants who refused to participate or travelled to a country where leishmaniasis is endemic were excluded [34].

### 2.3. Direct Interview

The agreed individuals were subject to interview concerning demographical data and history. Demographical data include name, gender, age, address, occupation, telephone or mobile number, and topographic surface area of home (escarpment or steep descending land or plateaus or flat land). History data including history of present or past lesion of CL. Unfortunately, participation of female subjects was very limited because of the custom and tradition in this region where the direct interview of female and foreigners is prohibited.

### 2.4. Clinical Examination

Skin surfaces and mucous membrane of oral and nasal cavities were carefully examined for any scar(s) or active lesions. Scars were assessed for the presence of six criteria of healed leishmaniasis lesions: no history of trauma, duration for > 2 weeks, round or oval shape, smooth surface, depressed scar, and pigment change, either hypo- or hyperpigmentation [35]. Likewise, active lesions were assessed for the presence of seven criteria: the first three listed above plus crater form ulcer with a raised border, smooth nodule, plaque, satellite lesion, and localized lymphadenopathy (subcutaneous nodule) [35–39]. All patients were referred for therapy in the primary health center. Patients with hoarseness of voice and aphonia were referred to hospital for direct and indirect laryngoscopic examination and management concerning MCL (Mucocutaneous Leishmaniasis). Patients with evidence of visceral leishmaniasis were referred to hospital for further management.

### 2.5. Leishmanin Skin Test (LST)

A mount of 0.1 ml of LST (Pasteur Institute; Iran) containing 6 × 106 L. major promastigotes was injected intradermally for all participants in the volar surface of the left forearm with a 27-Guage needle (BCG syringe). Readings were taken at 48 to 72 hours after injection using a ballpoint pen technique to determine the size of the indurations at the injection site [40]. An induration diameter of 5mm or greater was considered positive [41].

### 2.6. Data Analysis

All data were collected in data collection form and analyzed with standard tests including frequencies and percentages using the Statistical Package for the Social Sciences SPSS® version 22 (USA, Chicago). Proportions were compared by *χ*^2^ test or Fisher's exact test as appropriate.

## 3. Result

The study comprised 1165 participants; most of them were males (88%) while females were only 143 (12%). Participants with age less than 16 years were 451 (39%) followed by age group 16-30 years (36%), age group 31-45 years (14%), and participant over 45 years accounting for 11%. Participants living in plateau land were more than those living in escarpment land (60% and 40%, respectively). The physical examination revealed that overall percent of participants who had either scar, lesion, or both was 18.5% of the total population. Of them, 16 % had scar, 2% had lesion, and 0.5% had both scar and lesion. Scars in escarpment land were more prevalent than in plateaus land accounting for 31.7% (149/471 and 5.8% (40/694), respectively). Regarding gender it was more prevalent in females than males (23.1% (33/143) and 15.2% (156/1022), respectively). Participants less than 16 years were affected by scars than other age groups. Lesions were only found in escarpment land accounting for 4.7% (22/471). It was also more frequent in females than males (7.7% (11/143) and 1.1% (11/1022), respectively). No lesion was found in the age group 16-30 years while it accounted for 3.8% (17/451) in the age group < 16 years and it was equally distributed in the remaining age groups. Both scar and lesion were rarely found in the cases. More details about frequency are illustrated in Table 1.

The overall prevalence of LST positivity was 18.5% (215 out of 1165). The LST positivity reaction was prevalent within the participants who are living in escarpment land representing 34.8% (163/471) while those living in plateaus land represented only 7.5% with high statistically significant difference (P < 0.001). The survey revealed that LST was positive in 51.9% (98/189) of participants who had scar, 41% (9/22) of participants who had lesion, and 75% (3/4) of participants who had both lesion and scar. Only 11% (105/950) of participants who were free from lesion or scar positively reacted to LST with high statistically significant difference (P < 0.001). Prevalence of LST positivity reaction was 19.3% within males while within females it was only 12.6% with no statistically significant difference (P = 0.053). Regarding prevalence of LST positivity reaction within age groups, it ranged from 16% (72/451) within the children under 16 years of age to 26.4% (33/125) within the middle age and elderly participants. The increasing prevalence with age was statistically significant (P = 0.017) (Table 2).

**Figure 2:**
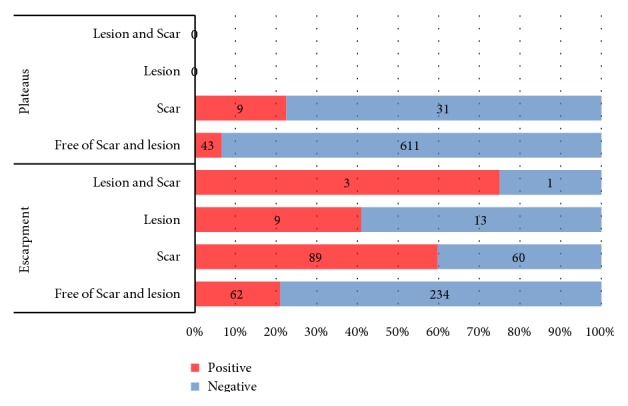
Prevalence of LST positivity in escarpment and plateaus areas.

As shown in Figure 2, no lesion or both scar and lesion were found in the plateau lands and only the Leishmanin skin test positivity accounted 23% and 7% for scar and among participants who were free of scar and lesion, respectively, whereas in escarpment land, the LST positivity reaction accounted for 21%, 60%, 41%, and 75% of participants who were free from scar and lesion, who had scar, who had lesion and who had both scar and lesion, respectively.

## 4. Discussion

The first suggestion that CL is prevalent in highlands of Yemen was reported since 1933 by Sarnelli [21]. He documented 5 cases of what he termed ‘leishmaniosi muco-cutanee' seen in Sana'a city. Later on, multiple documents confirmed that anthroponotic form of CL is the prevalent form in the highlands of Yemen [21, 22, 26, 27, 42–44]. Utmah district is a western part of these highlands consisted of escarpment land which is extremely steep and plateaus land which is less steep containing most of population. The current study is the first of its type in Yemen and revealed that CL is an endemic disease in Utmah district (18.5%), western highland in Yemen, and the infection is hyperendemic particularly in the escarpment land which accounted 34.8% of its entire population. In Yemen, no previous studies were done to evaluate the prevalence of leishmaniasis and all the documented studies based on data of patients attending clinics [5]. However, in Saudia Arabia, in Asir and Al-Baha mountainous, the same highland that extends in Yemen, Al-Zahrani et al. reported annual collected cases of CL that accounting 12/10000 person-year in Asir and 37/10000 person-year in Al-Baha.

In neighboring countries at the east Africa, where the biogeographically characteristics are similar, Lemma et al. [45] conducted LST survey in Silti district and found that the prevalence of LST positivity was 6.7% and represented 22.% and 44.2% in the towns Dessi and Karakore, respectively. These results were closely similar to the current study. Based on physical examination, other surveys in Ethiopia revealed lesions and scar with prevalence ranging from 4% to 10.7% for active lesion and 7% to 47% for scar of the past lesion [8–10, 13] which seem to be in agreement with the result of the present study. In contrast, these findings disagree with those reported in Sudan. Although Al-Hassan et al. reported similar result regarding scar and lesion, LST positivity was higher than that of the current study as well in Ethiopia. Kadaro et al. applied LST survey, along River Nile north of Khartoum where CL is prevalent, and found 94% of the population positively reacted; he reported that the lesions and scar represent 4% and 47% of population [13]. This may explain that, in Yemen, LST result represents prevalence L.* tropica*, the mainly causative agent of ACL in Yemen, while in Sudan LST may represent prevalence L.* tropica* and L.* major* the main causative agent of CL in Sudan in addition to the other circulating L. species of VL [13, 46, 47]. It is worthy to mention that more people in the Western Highlands, not only those with scars and lesions, have been infected by the parasite. The current survey revealed that the infection was prevalent at mountainous escarpment of the district. This finding is in agreement with that reported in Ethiopia and Saudi Arabia that confirmed that the slope is one of risk factor for leishmaniasis [8, 48]. Seid and Gadisa [49] evaluated environmental risk factors including elevation, temperature, soil, rainfall, and slope and reported that CL was more prevalent in escarpment land with slope greater than 4.6 degree. In Saudi Arabia, Al-Zahrani and Peters [43] also reported that CL was prevalent in escarpment area of western highland particularly in Asir and Al-Baha mountains; and no cases were detected in the lowland. Also the remaining risk factors, 10c-30c average temperature, 800mm 900mm annual rainfall, and 800m to 2800m elevation over sea level in the study area, match that reported by Seid in Ethiopia. Ministry of Public Health and Population in Yemen declares a recent CL outbreak in deferent regions including Utmah district. Yemen war may be attributed to be the cause of these reported outbreak CL; a number of endemic diseases emerged or become hyperendemic including CL in the region. In 2016, Du, Hotez [50] reported that the recent war in Middle East countries including Yemen may be another attributed factor that raised CL incidence to hyperendemic levels. During the survey, a number of construction works were going in the escarpment land of the district including making roads, dumb, and water barrier since 2013 that may create a favorable condition for life cycle of both reservoir and vector that subsequently cause raising infection in district [51].

Regarding the age group, the current study revealed that the positive reaction of LST raised with age starting by 16 % within participant under age 16 years and then increased gradually reaching 26.4% within participants over 45 years old. This result is in agreement with that of Weigle, Santrich [35] in Colombia, in which the author performed LST on 2656 participant and found that the LST positivity increased gradually with age starting from age group, 0-5 years old, with 0.6% reaching a constant level 21% at age 30 years. Weigle documented that the prevalence of infection increased with age reaching a constant level at 30 years [52]. In Mali, Traoré, Oliveira [53] performed LST survey in savanna forest and found LST positivity 2.7% in Kolondieba and 85.1% in Diema district [53]. In Diema district, where the infection was hyperendemic, the author reported that LST positivity increased steadily with age started by 13.8% within age 2-5y reaching 83.9% within the elderly 41–65 y. In Sudan, L.* major* is the prevalent L. species and the used LST is based on L. major. This fact may be the reason of high LST positivity in the Sudan, whereas in Yemen, L.* tropica* (ACL) is the most prevalent L. species causing the prevalence of reactive individuals to Leishmanin sensitization lower than that in the Sudan. Regarding the gender, number of published studies documented that less outdoor activity and dressing pattern of woman decrease the chance for parasite exposure and consequently decreased the prevalence of disease within the female [54, 55]. However, the gender comparisons might be biased and need to be done with caution. These findings agree with the findings of the current study; however the difference was not statistically significant. Participation of females in our study was limited and most of the invited females refused to participate which is related to the customs and traditions in the country. This can be considered as a limitation of the study. Another limitation of the study is the LST test as this test characteristically becomes positive 5-7 weeks after initiation of infection [56] and remained positive for 3–6 years following infection [55]. Moreover, sensitivity of LST in LCL (Localized Cutaneous leishmaniasis) patients varies ranging from 82% to 89% (98) while in MCL (Mucocutaneous Leishmaniasis) and DCL (Diffuse Cutaneous Leishmaniasis) patients it is usually 100% [57, 58]. However, sensitivity in patients with PKDL with concomitant visceral leishmaniasis is 11% while in those without concomitant visceral leishmaniasis it is 37% [59]. In addition, the LST does not differentiate between the different Leishmania species which have however different epidemiologies. Further studies are highly recommended to evaluate the associated risk factors. Authority awareness concerning a control program in the district includes treatment and prevention modalities. Public programs should be established to increase population awareness of the disease and how they can protect themselves.

## 5. Conclusion

According to the findings of the study, the following conclusion can be drawn:

Western highlands in Yemen are endemic areas for CL and the infection is hyperendemic in escarpment lands of the district particularly in individuals under 16 years and over 45 years old.

## Figures and Tables

**Table 1:** Distribution of leishmaniasis by district, gender, and age group.

		FREE OF LESION	SCAR	LESION	SCAR AND LESION

ALL SAMPLE (N= 1165)		950 (81.5)	189 (16.2)	22 (2)	4 (0.3)

DISTRICT	ESCARPMENT (N= 471)	296 (62.8)	149 (31.7)	22 (4.7)	4 (0.8)
	PLATEAUS (N= 694)	654 (94.2)	40 (5.8)	0 (0)	0 (0)

GENDER	MALE (N= 1022)	852 (83.4)	156 (15.2)	11 (1.1)	3 (0.3)
	FEMALE (N= 143)	98 (68.5)	33 (23.1)	11 (7.7)	1 (0.7)

AGE GROUP	<16Y (N= 451)	336 (74.5)	98 (21.7)	17 (3.8)	0 (0)
	16-30Y (N= 425)	371 (87.3)	52 (12.2)	0 (0)	2 (0.5)
	31-45Y (N= 164)	145 (88.4)	16 (9.8)	3 (1.8)	0 (0)
	>45Y (N= 125)	98 (78.4)	23 (18.4)	2 (1.6)	2 (1.6)

**Table 2:** Prevalence of LST positivity by district, PATIENT STATUS gender, and age group.

		LST POSITIVITY N (%)	*χ*2	P

ALL SAMPLE (N= 1165)		215 (18.5)		

DISTRICT	ESCARPMENT (N= 471)	163 (34.8)	138	< 0.001
	PLATEAUS (N= 694)	52 (7.5)		

PATIENT STATUS	FREE (N= 950)	105 (11.1)	190	< 0.001
	SCAR (N= 189)	98 (51.9)		
	LESION (N= 22)	9 (41)		
	SCAR AND LESION (N= 4)	3 (75)		

GENDER	MALE (N= 1022)	197 (19.3)	3.7	0.053
	FEMALE (N= 143)	18 (12.6)		

AGE GROUP	<16Y (N= 451)	72 (16)	10.52	0.017
	16-30Y (N= 425)	72 (16.9)		
	31-45Y (N= 164)	38 (23.2)		
	>45Y (N= 125)	33 (26.4)		

## Data Availability

This study is a part from Ph.D. thesis available at Department of Oral and Maxillofacial Surgery, Faculty of Dentistry, University of Khartoum, Khartoum, Sudan. The thesis will be available at http://khartoumspace.uofk.edu/handle/.
